# Network pharmacology integrated with experimental validation to explore the therapeutic role and potential mechanism of Epimedium for spinal cord injury

**DOI:** 10.3389/fnmol.2023.1074703

**Published:** 2023-01-30

**Authors:** Xuanhao Fu, Boyuan Ma, Mengmeng Zhou, Yuelin Cheng, Linyan Liu, Shunli Kan, Chengjiang Liu, Xinyan Zhao, Sa Feng, Haoqiang Zhu, Wei Hu, Zehua Jiang, Rusen Zhu

**Affiliations:** Department of Spine Surgery, Tianjin Union Medical Center, Tianjin, China

**Keywords:** spinal cord injury, Epimedium, network pharmacology, molecular docking, pharmacological mechanism

## Abstract

**Objective:**

Epimedium (EPI) is a common Chinese herb with neuroprotective effects against a variety of central nervous system disorders, especially spinal cord injury (SCI). In this study, we performed network pharmacology and molecular docking analyses to reveal the mechanism underlying EPI treatment of SCI, then validated its efficacy using animal models.

**Methods:**

The active ingredients and targets of EPI were screened by Traditional Chinese Medicine Systems Pharmacology (TCMSP) and their targets annotated on the UniProt platform. SCI-related targets were searched from OMIM, TTD, and GeneCards databases. We employed the STRING platform to construct a protein–protein interaction (PPI) network then visualized the results using Cytoscape (3.8.2) software. We also subjected key EPI targets to ontology (GO) and Kyoto Encyclopedia of Genes and Genomes (KEGG) enrichment analyses, then docked the main active ingredients with the key targets. Finally, we established an SCI rat model to evaluate efficacy of EPI in treating SCI and validate the effects of different biofunctional modules predicted by network pharmacology.

**Results:**

A total of 133 EPI targets were associated with SCI. GO terms and KEGG pathway enrichment results showed that EPI’s effect in treating SCI was significantly associated with inflammatory response, oxidative stress and the PI3K/AKT signaling pathway. Molecular docking results indicated that EPI’s active ingredients have a high affinity for the key targets. Results from animal experiments revealed that EPI not only markedly improved Basso, Beattie, and Bresnahan scores in SCI rats, but also significantly improved p-PI3K/PI3K and p-AKT/AKT ratio. Moreover, EPI treatment not only mediated a significant decrease in malondialdehyde (MDA) but also increased both superoxide dismutase (SOD), and glutathione (GSH). However, this phenomenon was successfully reversed by LY294002, a PI3K inhibitor.

**Conclusion:**

EPI improves behavioral performance in SCI rats through anti-oxidative stress, which may be mediated by activation of the PI3K/AKT signaling pathway.

## Background

Spinal cord injury (SCI) is a serious traumatic disease of the central nervous system, characterized by a high disability rate, resulting in abnormalities or loss of motor, sensory, and autonomic functions (Ahuja et al., 2017). SCI has a high annual incidence rate worldwide, about 40–80/1,000,000 people, a phenomenon that results in heavy economic burden on individuals, families, and society (Squair et al., 2016; Kumar et al., 2018; Alizadeh et al., 2019). Both primary and secondary injuries have been associated with SCI pathophysiology (Witiw and Fehlings, 2015). While primary injury refers to mechanical and direct injury to the spinal cord, secondary injury denotes several pathophysiological processes building on the primary injury, such as oxidative stress, inflammatory response, and apoptosis (Orr and Gensel, 2017; Zrzavy et al., 2021). Continuous and extensive cell death, coupled with tissue damage eventually cause permanent sensory and motor impairment (Liu et al., 2020). Although SCI currently lacks effective prevention and treatment modalities, numerous investigations have associated marked reduction of secondary injury with significant alleviation of disability rates in SCI patients (Song et al., 2021; Zhao et al., 2021). Therefore, promoting recovery of neurological function after SCI has great potential as a prevention and treatment modality for secondary injury.

Epimedium (EPI), a genus in family Berberidaceae with about 52 species, has been found to contain more than 260 compounds (Wu et al., 2003). In traditional Korean and Chinese clinics, EPI has been used for treatment of central nervous system-related diseases, such as Alzheimer’s disease, Parkinson’s disease, and multiple sclerosis, due to its neuroplasticity (Cho et al., 2017). In addition, EPI significantly restored motor function in rats after SCI, possibly by activating thePI3K/AKT signaling pathway (Li et al., 2019). On the other hand, Li demonstrated that EPI inhibited the mitochondrial apoptotic pathway to attenuate pro-inflammatory molecules and oxidative stress, which may be a key mechanism for enhanced motor recovery after SCI (Li et al., 2018). These evidences suggest that EPI is a very promising area of research in spinal cord repair and neurological recovery after SCI. To date, however, its necessitating pharmacological effects and underlying mechanisms of action in SCI remain unclear.

**Figure 1:**
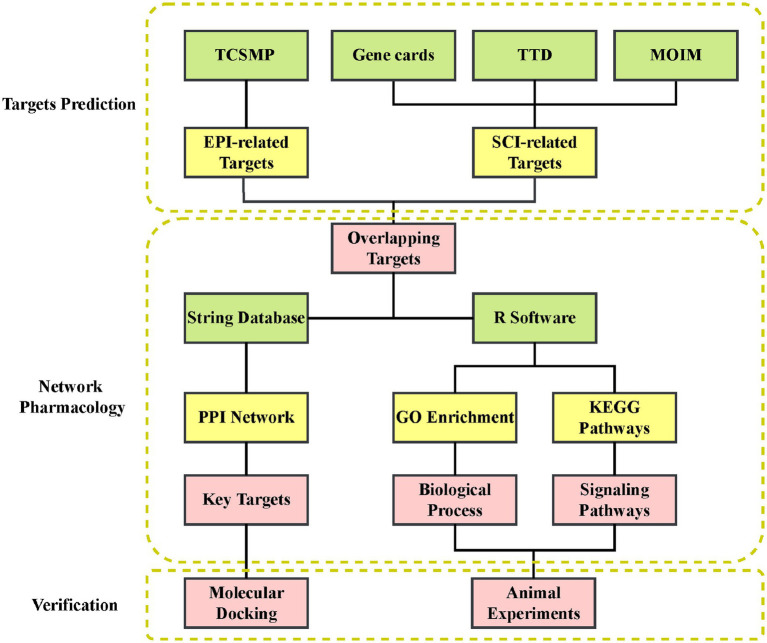
The analysis program flowchart of the study.

Network pharmacology, first proposed by Hopkins in the UK, is a technique used to systematically study the biological effects of complex drugs by integrating drug action with biological networks based on systems biology and multidirectional pharmacology (Hopkins, 2007). Numerous studies have shown that network pharmacology is a practical approach for systematic exploration of the relationship between herbal medicines and diseases (Wang et al., 2022; Zhou et al., 2022). In this study, we employed a compounds-targets network, based on pharmacology technique, in combination with molecular docking and animal experiments to reveal the effect of EPI and underlying mechanism of action in SCI. The overall flowchart of this study is shown in Figure 1.

## Materials and methods

### Screening of active ingredients and EPI targets

Traditional Chinese Medicine Systems Pharmacology (TCMSP) is a comprehensive pharmacological database of traditional Chinese medicine, comprising 3,311 targets and 29,384 components of 499 Chinese herbal medicines included in The Chinese Pharmacopoeia (Ru et al., 2014). In the process of drug screening, oral bioavailability (OB) is one of the most important pharmacokinetic parameters in the ADME characteristics (i.e., absorption, distribution, metabolism, excretion) of a drug, and is also an important indicator of the relative amount of drug delivered to the systemic circulation when administered orally. Drug-like properties represent the similarity of a compound to known drugs, and compounds with a high drug-like index (DL) are more likely to become drugs. We screened out active ingredients that met the various criteria, namely drug-likeness (DL) ≥ 0.18 and oral bioavailability (OB) ≥ 30% (Tsaioun et al., 2016; Sheng et al., 2021). Additionally, we obtained information related to the active ingredients ‘target proteins, then added gene names to the target protein using UniProt (https://www.Uniprot.org/; UniProt Consortium, 2018).

### Screening of SCI-related targets

We searched GeneCards (Stelzer et al., 2016), OMIM (Hamosh et al., 2005), and TTD (Wang et al., 2020) databases for SCI targets using “Spinal cord Injury” as a keyword (retrieved 17/11/2021), then processed the disease targets to obtain SCI-related targets. Finally, we analyzed the intersection between EPI-related and SCI-related targets using VennDiagram packages implemented in R software.

### Construction of a protein–protein interaction network

We employed the STRING database to build a PPI network, we uploaded the potential therapeutic targets of EPI for SCI to the STRING database and set the parameter to an interaction value >0.7 (Merico et al., 2010; Szklarczyk et al., 2017). We then used the CytoNCA plug-in in Cytoscape (3.8.2) software to obtain the key targets.

### Gene ontology and Kyoto encyclopedia of genes and genomes pathway enrichment analysis

Next, we explored the biological processes and signaling pathways associated with EPI in treatment of SCI by performing GO and KEGG pathway enrichment analyses using the clusterProfiler package in R (Kanehisa et al., 2008; Gene Ontology, 2015). Statistical significance threshold of enrichment analysis was set at *p* ≤ 0.05.

### Molecular docking

The interrelationships between key targets and active ingredients of EPI were visualized using the Sankey diagram. We also generated 3D structures of the active compounds using Chem Office software. These 3D structures were then downloaded from the PDB database, then the proteins dehydrated and dephosphorylated using PyMOL software (Rose et al., 2018). Autodock Vina was used to assess molecular docking (Trott and Olson, 2010).

### Establishment of SCI rat model

Adult female Sprague–Dawley rats (10-week-old) were purchased from Beijing Huafukang Biotechnology Co (license no: SCXK (Jing) 2019-008). Prior to experimental treatments, all rats were acclimated for 7 days in a controlled environment under a 12-h light/dark cycle with free access to food and drink. All animal experiments were approved by the Experimental Animal Ethics Committee of Tianjin People’s Hospital. Rats were anesthetized by intraperitoneal injection (ip), using 1% sodium pentobarbital (50 mg/kg), with the appropriate anesthetic effect confirmed by the pain reflex in the tail. After anesthesia, the rats were fixed in prone position, and the dorsum routinely prepared and disinfected with skin. The rats were subjected to laminectomy, at the Tenth thoracic vertebra (T10) level by incising each subcutaneous layer sequentially to fully expose the T10 segment of the spinal cord, which was not subject to impact injury. Next, a 10-gram impactor device (diameter: 2 mm) was lowered into the spinal cord from a height of 5 cm, to generate moderate spinal cord contusion (Yacoub et al., 2014). After modeling, the skin and muscle were stitched and the skin disinfected again with an iodophor. Rats in the sham group were subjected to laminectomy in the absence of contusion. To prevent infection, the rats were given penicillin, administered intramuscularly at a dose of 16 × 104 U per animal once daily for 3 consecutive days from the date of surgery. After surgery, the bladder was massaged twice a day, in the morning and evening, before its activity was restored.

### Drug administration

EPI was decocted twice (1 g/10 ml; 1.5 h each) in distilled water, then combined. The combined extracts were cooled to room temperature, and subsequently stored at −20°C in dry, sealed and contamination-free conditions until further use. LY294002 (154447-36-6), a PI3K inhibitor, was purchased from Aladdin, China and administered by intraperitoneal injection. After surgery, 40 rats were randomly divided into 4 groups and orally administered with EPI extract for 4 weeks. The treatments were as follows: sham group (sham surgery, 0.135 g/kg per day distilled water), SCI group (SCI, 0.135 g/kg per day distilled water), SCI + EPI group (SCI, 0.135 g/kg per day EPI), and SCI + EPI + LY group (SCI, 0.135 g/kg per day EPI, 1.2 mg/kg per day LY294002). Since oral administration is the main route of herbal drug delivery, we administered EPI orally to rats. The EPI dosage was adopted from the Chinese pharmacopeia clinical dose converted to body surface area (State Pharmacopoeia Commission of the PRC, 2015).

### Behavioral scores

Researchers, who were completely blinded to the study, assessed hind limb function of the rats by observing their hip, knee, and ankle joint walking movements for 5 min at1, 7, 14, 21, and 28 days post-operation. The function of the hind limbs was evaluated according to the Basso, Beattie, and Bresnahan (BBB) scores, where 0 and 21 considered complete paralysis and normal gait, respectively.

### Histopathological analysis

Spinal cord tissues were obtained from the rats on the 28^th^ day after operation. Firstly, ventricular perfusion with 0.9% physiological saline was performed under anesthesia, and 4% paraformaldehyde (PFA) injected to fix the tissue. Next, tissue from the center of the injury (1 cm) was obtained and immediately stored at-80°C to await western blot analysis. For H&E staining, spinal cord samples were fixed with 4% PFA, dehydrated in ethanol, dipped in wax, embedded in paraffin and cut into 6 μm sections using a microtome. The sections were then dewaxed and rehydrated, then stained with hematoxylin followed by eosin. The sections were permeabilized using xylene then visualized under a light microscope.

### Western blot assay

Spinal cord tissues, 1.0 cm around the center of the lesion, were collected from 3 individual rats and lysed in RIPA buffer (P0013B Beyotime, China) with a protease inhibitor cocktail (ST506 Beyotime, China). The tissue lysates were centrifuged at 12,000 rpm for 10 min at 4°C, and protein concentration determined using the double Bicinchoninic Acid (BCA) method (P0010, Beyotime, China). Equal concentrations of protein samples were separated on a 12% SDS polyacrylamide gel electrophoresis, then transferred to a polyvinylidene fluoride membrane. Next, the PVDF papers were blocked using skim milk for 1.5 h, then incubated overnight with the primary antibodies against GAPDH (1: 1000, Goodhere), phosphor-Akt (1: 3000, Proteintech Group), Akt (1: 2000, Affinity), phosphor-PI3K (1:2000, CST), and PI3K (1: 1000, Abcam) at 4°C. The membranes were washed 3 times using Tris-buffered saline Tween, then incubated with the secondary antibodies for 1 h at room temperature. Using ImageJ software, chemiluminescence was identified and measured.

### Statistical analysis

Data were first tested for normality using the Shapiro–Wilk normality test, then normally-distributed data subjected to one-way analysis of variance (ANOVA). Both non-normally distributed data and those with non-uniform variance were subjected to the Kruskal Wallis analysis. Statistical significance was set at *p* < 0.05.

## Results

### Active ingredients and targets of EPI

Using OB ≥ 30% and DL ≥ 0.18 as filtering conditions resulted in a total of 24 active EPI ingredients from the TCMSP database (Table 1). From these ingredients, we screened out 204 EPI-related targets using the TCMSP database and standardized by the UniProt database.

**Table 1:** OB and DL values of the 24 active compounds of EPI Molecule ID.

Mol ID	Molecule name	OB (%)	DL
MOL001510	24-Epicampesterol	37.57681789	0.71413
MOL001645	Linoleyl acetate	42.10076623	0.19845
MOL001771	Poriferast-5-en-3beta-ol	36.91390583	0.75034
MOL001792	DFV	32.76272375	0.18316
MOL003044	Chryseriol	35.85089483	0.27415
MOL003542	8-Isopentenyl-kaempferol	38.04433524	0.3948
MOL000359	Sitosterol	36.91390583	0.7512
MOL000422	Kaempferol	41.88224954	0.24066
MOL004367	Olivil	62.22859563	0.40642
MOL004373	Anhydroicaritin	45.41193421	0.43786
MOL004380	C-Homoerythrinan,1,6-didehydro-3,15,16-trimethoxy-, (3.beta.)-	39.13992598	0.49461
MOL004382	Yinyanghuo A	56.9573795	0.76747
MOL004384	Yinyanghuo C	45.67199685	0.50155
MOL004386	Yinyanghuo E	51.63212506	0.5474
MOL004388	6-hydroxy-11,12-dimethoxy-2,2-dimethyl-1,8-dioxo-2,3,4,8-tetrahydro-1H-isochromeno[3,4-h]isoquinolin-2-ium	60.64150904	0.65693
MOL004391	8-(3-methylbut-2-enyl)-2-phenyl-chromone	48.54449639	0.25066
MOL004394	Anhydroicaritin-3-O-alpha-L-rhamnoside	41.5834004	0.60981
MOL004396	1,2-bis(4-hydroxy-3-methoxyphenyl)propan-1,3-diol	52.31424958	0.22066
MOL004425	Icariin	41.5834004	0.61051
MOL004427	Icariside A7	31.90509191	0.85568
MOL000006	Luteolin	36.16262934	0.24552
MOL000622	Magnograndiolide	63.70888436	0.18833
MOL000098	Quercetin	46.43334812	0.27525

### Predicted SCI-related targets

**Figure 2:**
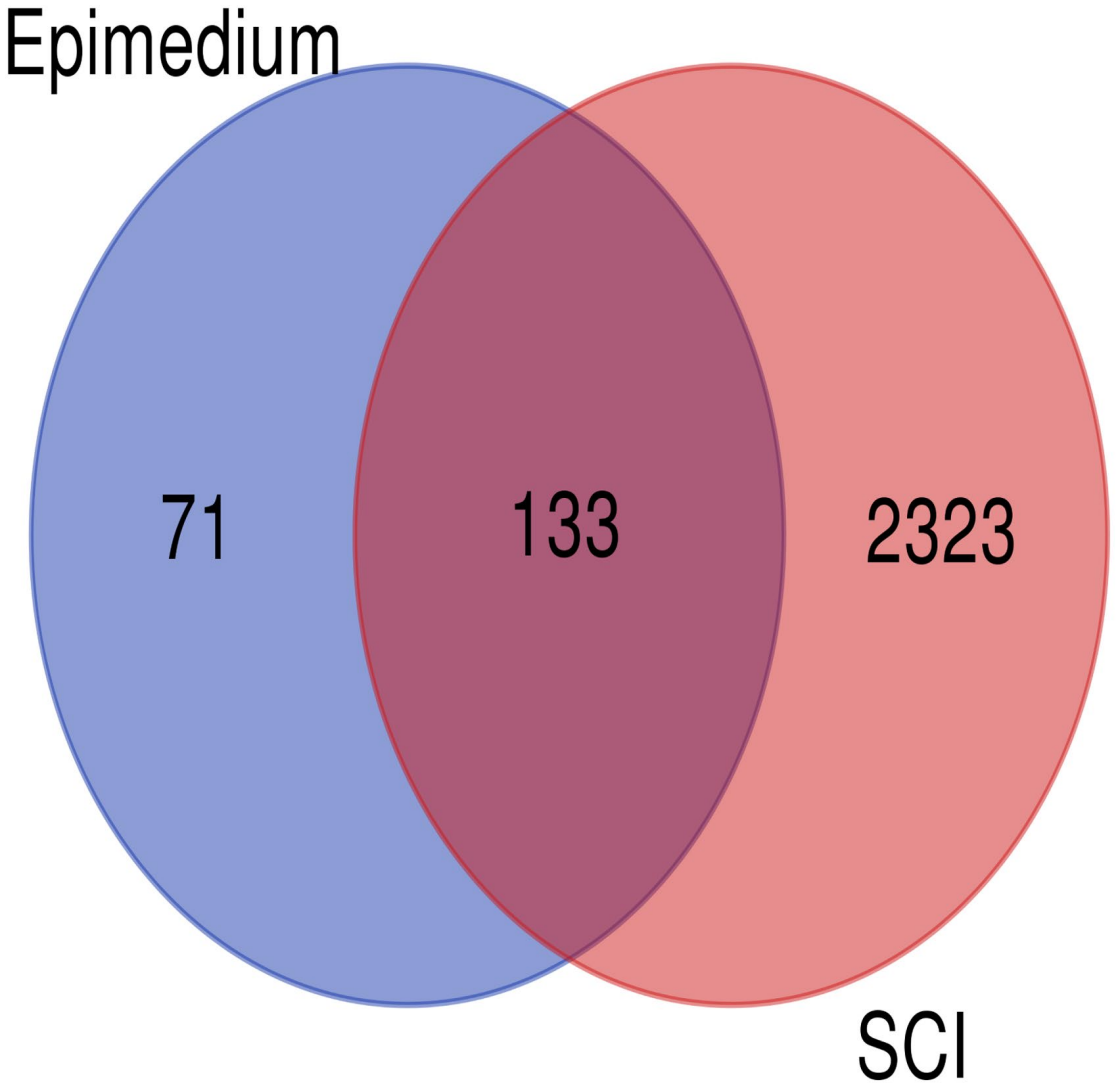
Venn diagram for EPI-and SCI-related targets.

Combining the three databases, coupled with deleting of repeated targets resulted in 2456 SCI-related targets by GeneCards, OMIM, and TDD databases. Analysis of the intersection between 204 EPI-related and 2,456 and SCI-related targets, resulted in 133 potential targets of EPI for SCI (Figure 2).

### Construction of component-target network of EPI for SCI

**Figure 3:**
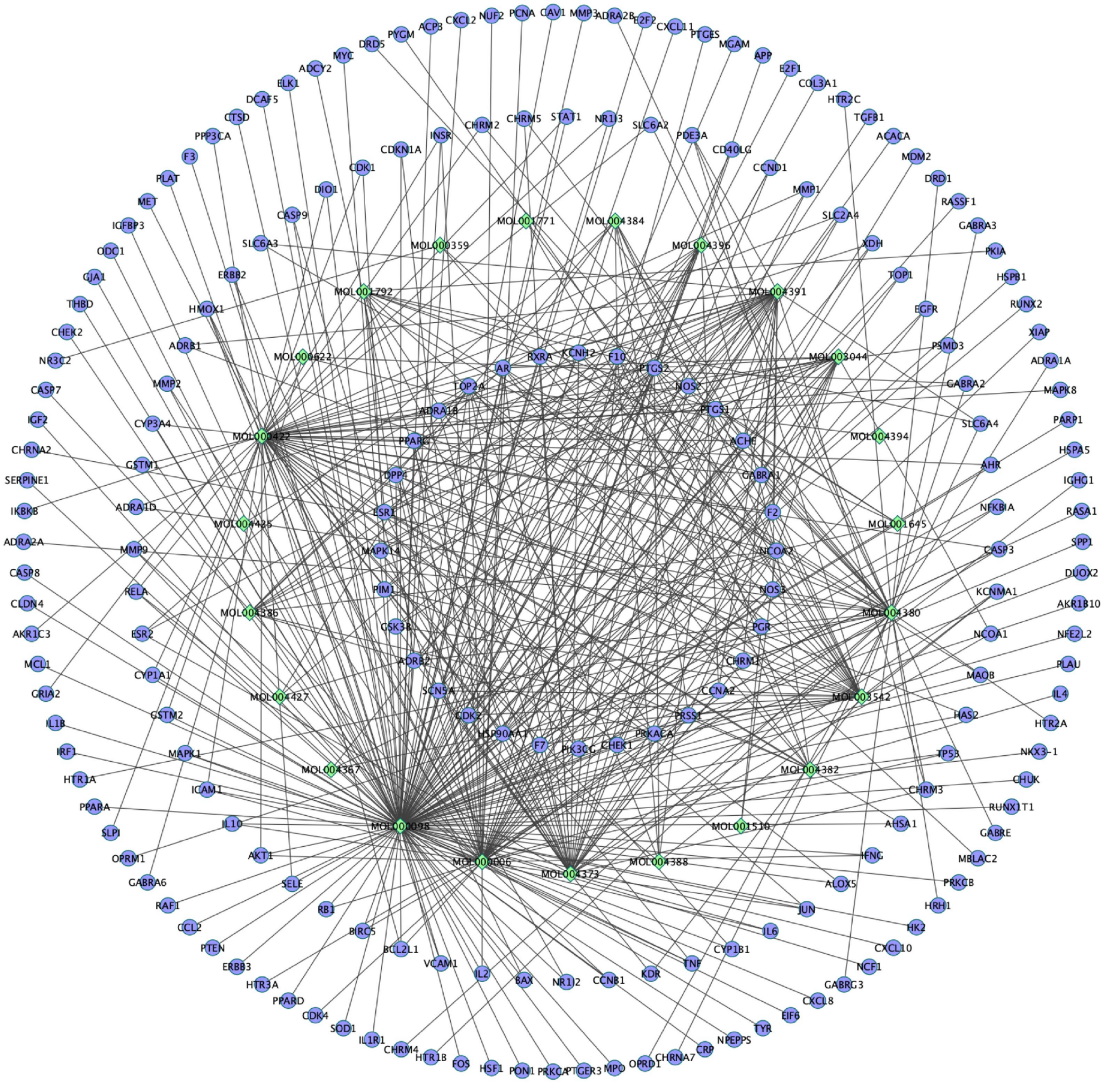
The compounds-targets network diagram of EPI to treat SCI. Rhombic nodes represent active ingredients in EPI, circular nodes represent 133 potential targets of EPI for SCI.

Based on 133 potential targets and 24 active ingredients, the compounds-targets network diagram of EPI to treat SCI was plotted (Figure 3). Collectively, these results suggested that EPI may act synergistically through multiple components and multiple targets during treatment of SCI.

### PPI network

**Figure 4:**
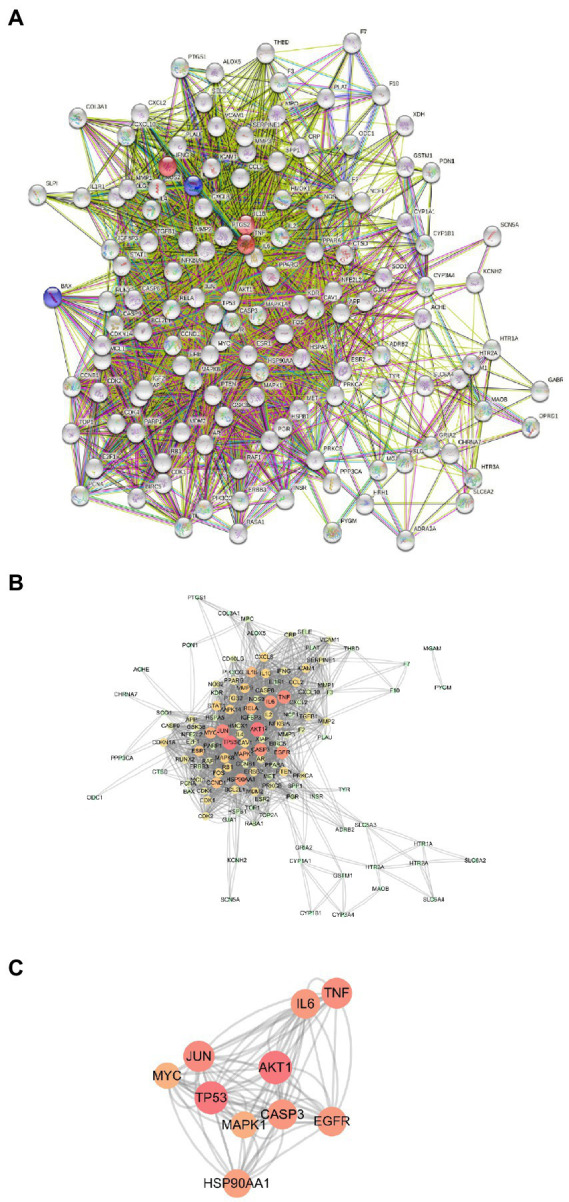
PPI Network Analysis. **(A)** PPI network of 133 potential targets of EPI for SCI. **(B)** PPI network constructed with Cytoscape software (The darker the node color, the higher the number of connected proteins). **(C)** The key targets of EPI for SCI obtained.

Next, we used the STRING program to construct a PPI network of the 133 potential EPI targets for SCI (Figure 4A). The resulting network contained 126 nodes and 1968 edges. The top 10 genes in the Degree algorithm were AKT1, TP53, JUN, TNF, CASP3, EGFR, IL6, HSP90AA1, MAPK1, and MYC, which can affect a wider range of proteins (Figures 4B,C).

### GO terms and KEGG pathway enrichment

**Figure 5:**
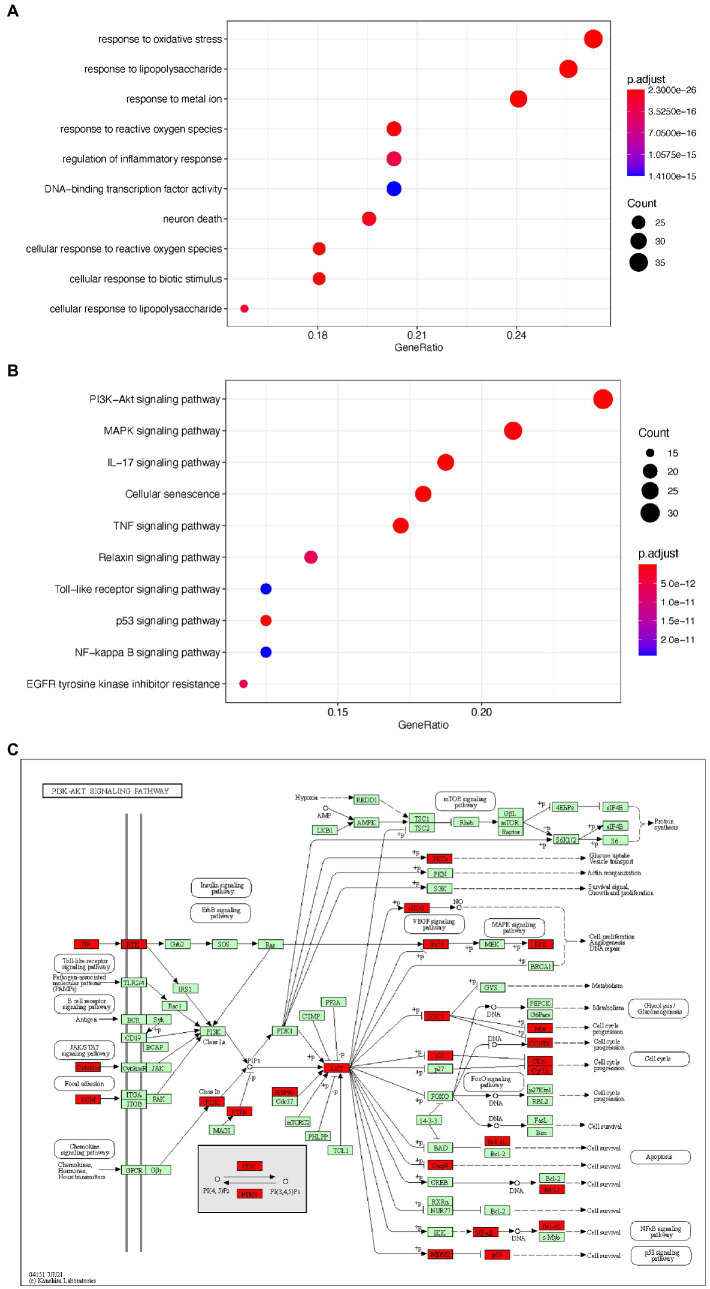
The GO and KEGG enrichment analysis of the potential targets of EPI for SCI. **(A)** the top 10 enriched GO terms. **(B)** The top 10 enriched KEGG signaling pathways. **(C)** PI3K/AKT signaling pathway map (Green and red are all genes on the PI3K signaling pathway, and red represents the targets of EPI for SCI). PI3K/AKT signaling pathway map is mined here from https://www.kegg.jp/kegg/mapper/.

To elucidate the potential mechanism underlying EPI’s action in SCI, we used the clusterProfiler package to perform GO and KEGG pathway enrichment analyses on the 133 potential EPI targets. The top 10 GO items and enriched KEGG pathways are shown in Figures 5A,B while detailed information is shown in Tables 2 and 3. In addition, KEGG analysis indicated that the PI3K/AKT signaling pathway was enriched with the most genes (Figure 5C). These results suggested that EPI may be regulating SCI repair by modulating oxidative stress, inflammatory response, neuron death, and PI3K/AKT signaling pathways.

**Table 2:** Top 10 items of Gene Ontology (GO) enrichment analysis.

ID	Description	*p* value	*p*. adjust	Count
GO:0006979	Response to oxidative stress	1.44E-26	1.17E-23	35
GO:0032496	Response to lipopolysaccharide	5.65E-30	2.30E-26	34
GO:0010038	Response to metal ion	4.27E-26	2.89E-23	32
GO:0000302	Response to reactive oxygen species	3.10E-25	1.40E-22	27
GO:0050727	Regulation of inflammatory response	1.96E-18	3.06E-16	27
GO:0051090	DNA-binding transcription factor activity	1.11E-17	1.41E-15	27
GO:0070997	Neuron death	3.79E-19	7.34E-17	26
GO:0034614	Cellular response to reactive oxygen species	1.11E-24	4.53E-22	24
GO:0071216	Cellular response to biotic stimulus	8.91E-21	2.41E-18	24
GO:0071222	Cellular response to lipopolysaccharide	1.40E-18	2.28E-16	21

**Table 3:** The enriched 10 possible related pathways for SCI.

ID	Description	*p* value	*p*. adjust	Count
hsa04151	PI3K-Akt signaling pathway	1.95E-15	2.06E-14	31
hsa04010	MAPK signaling pathway	5.39E-14	3.97E-13	27
hsa04657	IL-17 signaling pathway	3.58E-23	2.18E-21	24
hsa04218	Cellular senescence	1.64E-16	2.35E-15	23
hsa04668	TNF signaling pathway	1.30E-18	2.88E-17	22
hsa04926	Relaxin signaling pathway	1.11E-12	6.57E-12	18
hsa04620	Toll-like receptor signaling pathway	4.70E-12	2.43E-11	16
hsa04115	p53 signaling pathway	1.40E-14	1.26E-13	16
hsa04064	NF-kappa B signaling pathway	4.70E-12	2.43E-11	16
hsa01521	EGFR tyrosine kinase inhibitor resistance	9.34E-13	5.68E-12	15

### Molecular docking

**Figure 6:**
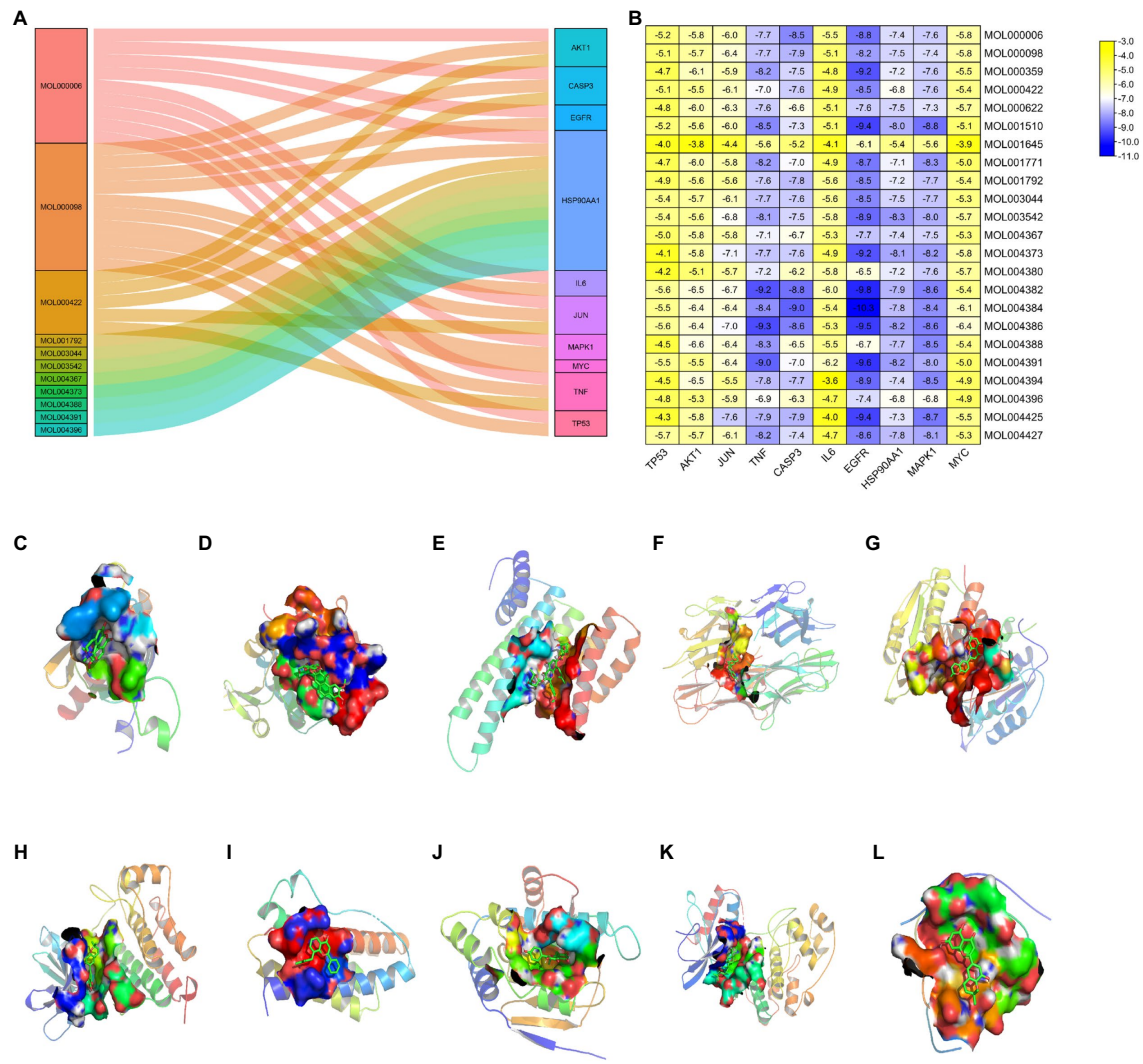
Molecular docking between active ingredients and key targets. **(A)** Sankey diagram of “compounds-targets.” **(B)** Heatmap of the scores of molecular docking in this study. The darker the color, the better the binding of active ingredient to the key target. **(C)** MOL004388 to AKT1. **(D)** MOL004427 to TP53. **(E)** MOL004425 to JUN. **(F)** MOL004386 to TNF. **(G)** MOL004384 to CASP3. **(H)** MOL004384 to EGFR. **(I)** MOL004391 to IL6. **(J)** MOL003542 to HSP90AA1. **(K)** MOL001510 to MAPK1. **(L)** MOL000006 to MYC.

To validate results of the network pharmacology, we used molecular docking to evaluate the interaction between active ingredients and key targets. A resulting Sankey diagram revealed that the 10 key targets were related to active EPI ingredients. Particularly, quercetin was associated with 10 targets, luteolin corresponded to 9 targets, kaempferol corresponded to 5 targets, while other active ingredients corresponded to 1 target each (Figure 6A). Molecular docking scores suggested that most of active ingredients bind core target proteins with binding energies below-5.0, indicating that the target specifically binds the compound (Figure 6B). The active ingredients and targets with the highest scoring free binding energy are shown in corresponding molecular docking diagrams (Figures 6C–L). Collectively, these results revealed that active ingredients have a strong affinity for the identified targets.

### Effect of EPI on motor performance and tissue damage in rats with SCI

**Figure 7:**
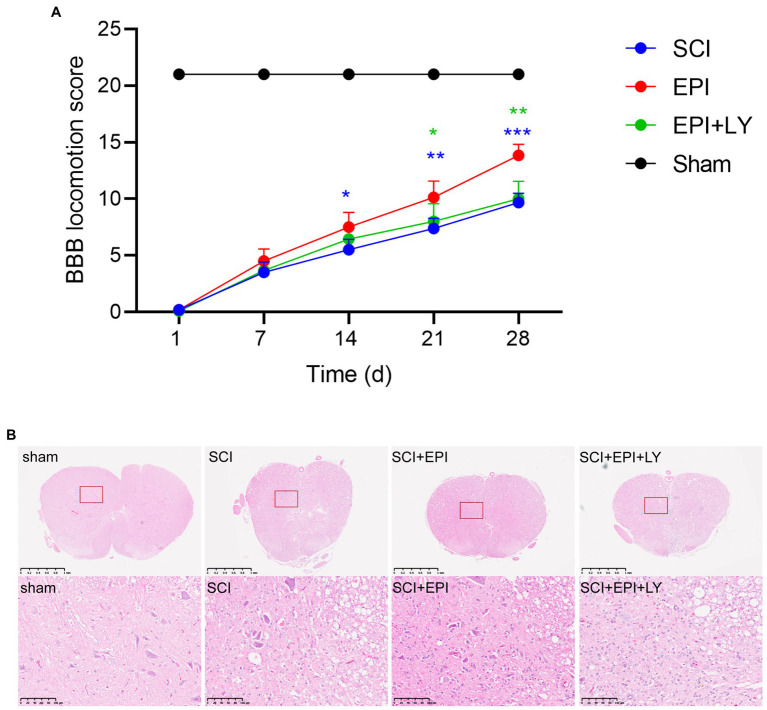
EPI enhances behavioral performance and tissue repair in SCI rats (N = 3 per group). **(A)** Use BBB score to evaluate the recovery of motor function in sham group, SCI group, SCI + EPI group, and SCI + EPI + LY group, respectively, in 3, 7, 14, 21, 28 days (N = 10 per group). **(B)** HE-stained transverse sections of spinal cords from sham group, SCI group, SCI + EPI group, and SCI + EPI + LY group (N = 3 per group). * means *p* < 0.05, ** means *p* < 0.01, and *** means *p* < 0.001.

To assess whether EPI promotes recovery of motor function in SCI rats, we first established an SCI rat model then analyzed the resulting BBB scores to understand behavioral performance. Rats in the sham group exhibited a BBB motor score of 21 at each postoperative time point (1, 7, 14, and 28 days), with no evidence of hindlimb motor dysfunction (Figure 7A). Conversely, rats in SCI, SCI + EPI, and SCI + EPI + LY groups exhibited severe motor dysfunction at 1 day post-operation, as evidenced by a score close to 0. It continued to rise during the experimental period but remained significantly lower in the SCI group, EPI, and EPI + LY group than in the sham group at all time points. Rats in the SCI + EPI group exhibited significantly higher BBB scores at 14, 21, and 28 d after SCI, relative to their SCI counterparts, while those in the SCI + EPI had a significantly higher behavior compared to those in the SCI + EPI + LY group at 21 and 28 d after SCI. There were no significant differences between rats in the SCI and SCI + EPI + LY groups at individual time points. We further analyzed the effect of EPI on spinal cord histopathology using H&E staining and found that rats in the SCI group exhibited significantly higher congestion, structural disorders, and structural damage than those in the sham group (Figure 7B). Notably, EPI treatment significantly improved the above-mentioned protective effect pathological symptoms, consistent with results of motor assessment. Overall, these results indicated that EPI not only reduced the degree of edema and cavitation of spinal cord tissue, but also improved motor function in the hind limbs of rats after SCI.

### PEI regulates PI3K/AKT pathway signaling after SCI

**Figure 8:**
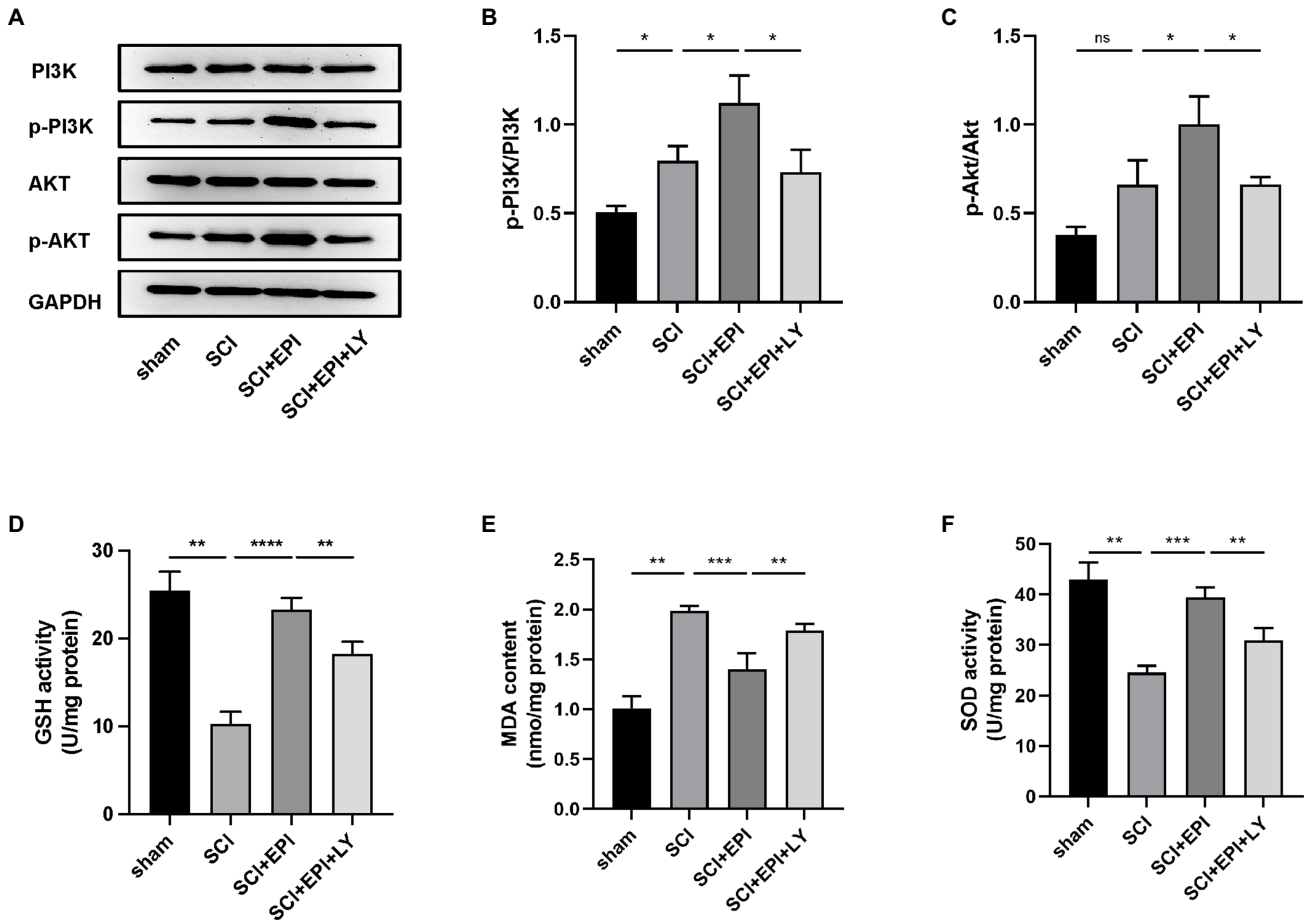
Investigation of the PI3K/AKT signaling pathway in the antioxidant effect of EPI (N = 3 per group). **(A)** The expressions of PI3K, p-PI3K, AKT, and p-AKT were detected by Western blot assays. **(B,C)** Quantitative analysis of western blot assays. **(D–F)** The concentration of GSH, MDA, and SOD in sham group, SCI group, SCI + EPI group, and SCI + EPI + LY. * means *p* < 0.05, ** means *p* < 0.01, and *** means *p* < 0.001.

Network pharmacology analysis results revealed that EPI treats SCI by modulating the PI3K/Akt signaling pathway. To verify the importance of the PI3K/Akt signaling pathway in SCI treatment, we analyzed expression levels of p-PI3K, PI3K, p-Akt, and Akt proteins in the spinal cord tissue of rats after EPI treatment. We also used LY294002, a specific PI3K inhibitor, to inhibit PI3K/Akt signaling pathway to further validate the importance of EPI for SCI recovery. Western blots revealed that the p-PI3K/PI3K ratio was significantly higher in the spinal cord of SCI rats relative to those in the sham group, whereas the p-AKT/AKT ratio was not significantly different between the groups (Figures 8A–C). p-PI3K/PI3K and p-AKT/AKT ratio significantly increased after EPI treatment, compared with the SCI group, and these increases were significantly inhibited by the LY294002 treatment. Overall, these results indicated that EPI activated the PI3K/AKT signaling pathway in SCI rats, which was consistent with results of network pharmacology analysis.

### EPI alleviates oxidative stress by activating the PI3K/AKT signaling pathway after SCI

Next, we investigated whether EPI affects the ability to resist oxidative stress through PI3K/AKT pathway by analyzing the effect of EPI on spinal cord tissue in SCI rats. Experimental results revealed that GSH and SOD activities were significantly lower while MDA activity was significantly higher in spinal cord tissue of SCI rats, relative to those in the sham group, consistent with previous results by Li et al. (2022; Figures 8E,F). Further, rats in the SCI + EPI group had significantly higher GSH activities than their SCI counterparts, whereas those in the SCI + EPI + LY group had lower activities than their SCI + EPI counterparts (Figure 8D). Rats in the SCI + EPI group exhibited significantly lower MDA levels than those in the SCI group while those in the SCI + EPI + LY group were higher than in the SCI + EPI group (Figure 8E). However, SOD expression was downregulated in the SCI + EPI group than in the SCI + EPI group, and higher than that in the SCI + EPI + LY group (Figure 8F). These results indicated that LY294002 affected the ability of EPI to reduce oxidative stress by supplementing the role of EPI in activating PI3K/AKT signaling pathway. Collectively, these results indicated that EPI alleviates oxidative stress in the spinal cord tissue of SCI rats by activating the PI3K/AKT signaling pathway.

## Discussion

SCI, a major traumatic disorder of the central nervous system that can lead to permanent motor and sensory impairment, has been shown to affect approximately 930,000 people worldwide each year (Disease et al., 2018). In recent years, several studies have revealed the active ingredients of EPI and demonstrated its neuroprotective effects in SCI (Tohda and Nagata, 2012; Jia et al., 2019). Traditional research on drugs for SCI is limited to a one-drug, one-target model, but SCI is a multi-target and multifactorial disease and it is difficult to achieve ideal therapeutic results based on a single target alone. By collecting drug and disease information from public databases, network pharmacology explains the molecular mechanism of the active ingredients of EPI in the treatment of SCI from multi-component, multi-target, and multi-pathway. Thus, we employed this new approach, coupled with animal experiments, to investigate the protective effect and mechanism underlying EPI action in the treatment of SCI. Analysis of biological activities, core therapeutic targets, and potential mechanisms underlying EPI’s action in SCI revealed 24 active ingredients and 133 potential therapeutic targets. In addition, PPI network analysis revealed 10 key EPI targets, namely Akt1, Tp53, Jun, Tnf, Casp3, Egfr, Il6, Hsp90aa1, Mapk1, and Myc, which were closely related to SCI development. These proteins are regarded as core proteins that may play an essential role in the therapeutic effect of EPI on SCI. TNF activity increases, participating in the inflammatory and injury cascade response, exacerbating ischemia and hypoxia in the injury area, and inducing axonal damage and degeneration after SCI (Yan et al., 2001; Pineau and Lacroix, 2007). Recent studies have shown that conditional elimination of TNF in macrophages and neutrophils improves recovery of motor function and reduces the area of injury in mice with SCI (Ellman et al., 2020). Inflammatory mediators such as TNF-α and IL6 mediate the recruitment of inflammatory cells to the injury site. Targeting these cytokines may be a potential strategy to reduce secondary injury in SCI (Esposito and Cuzzocrea, 2011; Kim et al., 2021). Molecular docking results revealed that luteolin and Yinyanghuo E had high affinity and were bound to IL6 and TNF, respectively. These results affirmed the role played by IL6 and TNF in EPI for SCI.

In the present study, GO terms indicated that EPI could treat SCI by modulating oxidative stress, inflammatory response, and neuron death, as well as other biological functional modules. The targets were significantly enriched in PI3K/Akt, MAPK, and IL-17 signaling pathway signaling pathways, which are closely related to the SCI signaling pathway. Among them, the PI3K-AKT signaling pathway appears to be the most enriched in SCI-related signaling pathways and seems to be the most important pathway for the treatment of SCI. Previous studies have shown that the PI3K/AKT signaling pathway plays important roles in secondary SCI, such as anti-inflammatory, anti-apoptotic, and regulating glial scar formation (Dong et al., 2014; Chen et al., 2016; Li et al., 2020). In addition, Wang showed that activation of the PI3K/Akt signaling pathway could inhibit excessive oxidative stress (Wang et al., 2020). These reports were consistent with our prediction which indicated that the PI3K/AKT signaling pathway is not only essential for EPI treatment of SCI but may also be regulating the observed antioxidant activity.

Phosphatidylinositol 3 kinases (PI3Ks), which belong to the lipid kinase family, are activated by various stimuli to produce phosphatidylinositol-3-phosphate (PIP3) which subsequently directly activates conversion of threonine proteins (AKT) into phosphorylated proteins (p-AKT) by stimulating serine-dependent activase (PDK; Heras-Sandoval et al., 2014). Notably, PI3K is required for the activation of Akt, a member of the serine/threonine protein activase family (Heras-Sandoval et al., 2014). In the present study, we administered the EPI to rats and found that EPI not only improved motor function, and reduced tissue damage, but also significantly improved p-PI3K/PI3K and p-AKT/AKT ratio after SCI. In addition, LY294002 decreased the ability of EPI to improve motor and tissue damage and reduced p-PI3K/PI3K and p-AKT/AKT ratio. Collectively, these findings suggested that EPI exerts a protective effect after SCI through the PI3K/Akt signaling pathway.

Previous studies have shown that oxidative stress markedly exacerbates both SCI and diabetic neuropathy (Fernyhough and McGavock, 2014; Zrzavy et al., 2021). Moreover, oxidative stress has not only been reportedly linked to the pathogenesis of SCI, but also to levels of oxidative markers such as SOD, GSH, and MDA in the spinal cord tissue (Li et al., 2022). Results of the present study showed that treatment with EPI extracts significantly increased SOD and GSH and decreased MDA, demonstrating that EPI may exert SCI protective effects through antioxidant effects. Furthermore, PI3K inhibitors partially reversed the antioxidant capacity of EPI extracts, suggesting that the PI3K/AKT signaling pathway may be a potential underlying mechanism EPI against oxidative stress after SCI.

This study had some limitations. Firstly, the data obtained from the online databases were based on researched and anticipated material, therefore unverified and undocumented active ingredients or genes might not be considered our study. Secondly, in addition to the biological modules validated in this experiment, EPI may also exert protective effects through other pathways. Further research explorations are needed to validate each biofunctional module. Thirdly, the basis of EPI’s treatment of SCI is that it contains a large number of compounds, and we will conduct more experiments to explore the role of key compounds in EPI.

## Conclusion

In summary, we employed a combination of network pharmacology and animal experiments to effectively reveal the biological-molecular mechanisms underlying EPI’s action in treatment of SCI. Particularly, our results indicated that EPI ameliorates SCI in rats through antioxidant effects, which may be related to the activation of the PI3K/AKT signaling pathway. These findings affirm EPI’s significance as a potential therapy for treatment of SCI.

## Data availability statement

The original contributions presented in the study are included in the article/supplementary material, further inquiries can be directed to the corresponding author.

## Ethics statement

The animal study was reviewed and approved by Experimental Animal Ethics Committee of Tianjin People’s Hospital.

## Author contributions

XF, BM, MZ, and RZ designed the experiments and analyzed data. YC, LL, SK, CL, and ZJ performed the experiments. XZ, SF, HZ, and WH wrote the paper. All authors contributed to the article and approved the submitted version.

## Funding

This study was supported by Tianjin Key Medical Discipline (Specialty) Construction Project (TJYXZDXK-064B), Foundation of Tianjin Union Medical Center (2019JZPY01), and Tianjin Health Commission Science and Technology Project (ZC20225 and KJ20061).

## Conflict of interest

The authors declare that the research was conducted in the absence of any commercial or financial relationships that could be construed as a potential conflict of interest.

## Publisher’s note

All claims expressed in this article are solely those of the authors and do not necessarily represent those of their affiliated organizations, or those of the publisher, the editors and the reviewers. Any product that may be evaluated in this article, or claim that may be made by its manufacturer, is not guaranteed or endorsed by the publisher.
