# Corrigendum: Network pharmacology integrated with experimental validation to explore the therapeutic role and potential mechanism of Epimedium for spinal cord injury

**DOI:** 10.3389/fnmol.2023.1257836

**Published:** 2023-07-26

**Authors:** Xuanhao Fu, Boyuan Ma, Mengmeng Zhou, Yuelin Cheng, Linyan Liu, Shunli Kan, Chengjiang Liu, Xinyan Zhao, Sa Feng, Haoqiang Zhu, Wei Hu, Zehua Jiang, Rusen Zhu

**Affiliations:** Department of Spine Surgery, Tianjin Union Medical Center, Tianjin, China

**Keywords:** spinal cord injury, Epimedium, network pharmacology, molecular docking, pharmacological mechanism

In the published article, there was an error in affiliation “1”. Instead of “Tianjin People's Hospital”, it should be “Tianjin Union Medical Center”.

Due to the change of the name of our institution from “Tianjin People's Hospital” to “Tianjin Union Medical Center”, we propose to correct the original institution name.

The authors apologize for this error and state that this does not change the scientific conclusions of the article in any way. The original article has been updated.

